# Supplementary material for: Attitudes and intentions of parents towards the COVID-19 vaccine for their children at a special moment of the pandemic
Source: PeerJ. 2024 Sep 25;12:e18056. doi: 10.7717/peerj.18056 (PMC11438426; doi:10.7717/peerj.18056)
Supplement: Supplemental Information 3 [file peerj-12-18056-s003.docx]

| **Koronavirüs Anksiyete Ölçeği** | | | | | |
| --- | --- | --- | --- | --- | --- |
| *Son 2 hafta boyunca aşağıdaki durumları ne sıklıkta yaşadınız?* | Hiç | Nadir, bir veya iki günden az | Birkaç gün | 7 günden fazla | Son 2 haftada neredeyse her gün |
| 1. Koronavirüs ile ilgili haberleri okuduğumda veya dinlediğimde başım döndü, sersemlemiş¸ veya baygın hissettim. |  |  |  |  |  |
| 2. Koronavirüs hakkında düşündüğüm için uykuya dalma veya uykuda kalma konusunda sorun yaşadım. |  |  |  |  |  |
| 3. Koronavirüs hakkında düşündüğümde veya bilgiye maruz kaldığımda felç¸ olmuş¸ veya donmuş¸ gibi hissettim. |  |  |  |  |  |
| 4. Koronavirüs hakkında düşündüğümde veya bilgiye maruz kaldığımda yemek yemeye ilgimi kaybettim. |  |  |  |  |  |
| 5. Koronavirüs hakkında düşündüğümde veya bilgiye maruz kaldığımda mide bulantısı hissettim veya mide problemleri yaşadım. |  |  |  |  |  |
