# Supplementary material for: Attitudes and intentions of parents towards the COVID-19 vaccine for their children at a special moment of the pandemic
Source: PeerJ. 2024 Sep 25;12:e18056. doi: 10.7717/peerj.18056 (PMC11438426; doi:10.7717/peerj.18056)
Supplement: Supplemental Information 4 [file peerj-12-18056-s004.docx]

| **Covid-19 Aşısına Yönelik Tutumlar Ölçeği** | | | | | |
| --- | --- | --- | --- | --- | --- |
| *Lütfen aşağıdaki durumlara katılma düzeyinize uygun durumunuzu işaretleyiniz.* | Kesinlikle katılmıyorum | Katılmıyorum | Ne katılıyorum ne katılmıyorum | Katılıyorum | Kesinlikle katılıyorum |
| 1.Ailemdekilerin bu hastalıkla ilgili geliştirilecek/geliştirilen aşıyı olmasını isterim. |  |  |  |  |  |
| 2.İlk fırsatta bu hastalıkla ilgili geliştirilecek/geliştirilen aşıyı olmak isterim. |  |  |  |  |  |
| 3. Bence herkes bu hastalıkla ilgili geliştirilecek/geliştirilen aşıyı yaptırmalı. |  |  |  |  |  |
| 4. Geliştirilecek/geliştirilen aşı hakkında yapılan açıklamalara güveniyorum. |  |  |  |  |  |
| 5.Geliştirilecek/geliştirilen aşı hastalığın bulaşmasına neden olabilir. |  |  |  |  |  |
| 6.Geliştirilecek/geliştirilen aşının koruyucu etkisinin olmayacağını/olmadığını düşünüyorum. |  |  |  |  |  |
| 7. Geliştirilecek/geliştirilen aşı tehlikelidir. |  |  |  |  |  |
| 8.Geliştirilecek/geliştirilen aşının etkililiği yeterince test edilmeyeceğini/edilmediğini düşünüyorum. |  |  |  |  |  |
| 9.Aşı olmadan da salgını atlatabileceğimi düşünüyorum. |  |  |  |  |  |
